# Environmental change mediates mate choice for an extended phenotype, but not for mate quality

**DOI:** 10.1111/evo.13091

**Published:** 2016-11-02

**Authors:** Megan L. Head, Rebecca J. Fox, Iain Barber

**Affiliations:** ^1^ Department of Neuroscience, Psychology and Behaviour, College of Medicine, Biological Sciences and PsychologyUniversity of LeicesterUniversity RoadLeicesterLE1 7RHUnited Kingdom; ^2^ Division of Evolution, Ecology, and Genetics, Research School of BiologyAustralian National UniversityCanberraACT2600Australia; ^3^ School of Life SciencesUniversity of Technology SydneyUltimoNSW2007Australia

**Keywords:** Behavioral plasticity, extended phenotype, mate choice, multiple cues, nest, stickleback

## Abstract

Sexual cues, including extended phenotypes, are expected to be reliable indicators of male genetic quality and/or provide information on parental quality. However, the reliability of these cues may be dependent on stability of the environment, with heterogeneity affecting how selection acts on such traits. Here, we test how environmental change mediates mate choice for multiple sexual traits, including an extended phenotype–‐the structure of male‐built nests – in stickleback fish. First, we manipulated the dissolved oxygen (DO) content of water to create high or low DO environments in which male fish built nests. Then we recorded the mate choice of females encountering these males (and their nests), under either the same or reversed DO conditions. Males in high DO environments built more compact nests than those in low DO conditions and males adjusted their nest structure in response to changing conditions. Female mate choice for extended phenotype (male nests) was environmentally dependent (females chose more compact nests in high DO conditions), while female choice for male phenotype was not (females chose large, vigorous males regardless of DO level). Examining mate choice in this dynamic context suggests that females evaluate the reliability of multiple sexual cues, taking into account environmental heterogeneity.

Mate choice is expected to select for traits that are a reliable indicator of mate quality (Johnstone 1995; Kokko et al. 2006). But when habitats vary either spatially or temporally, sexual cues that were selected for under one set of environmental conditions may not match the new environment and may become costly maladaptions (Candolin et al. 2007). As a consequence, females may make mate choice decisions based on unreliable indicators of male quality (Wong et al. 2007; Candolin et al. 2016). This context‐dependent validity of sexual cues can lead to environmentally dependent mate choice preferences (Heuschele et al. 2009) with consequences for the strength or direction of selection within a population (Jävenpää and Lindström 2004; Candolin et al. 2007; Wong et al. 2007; Candolin et al. 2016) and, ultimately, for population dynamics (Candolin et al. 2016) and levels of genetic variation in fitness within populations (Huang et al. 2015). For example, enhanced water turbidity reduces reliance on visual traits for assessing male quality in gobies (Jävenpää and Lindström 2004; Michelangeli et al. 2015) and in sticklebacks (Wong et al. 2007; Candolin et al. 2007; Heuschele et al. 2009; Candolin et al. 2016), leading to increased energetic investment in courtship by males and reliance on alternative traits (e.g., olfactory cues) by females (Michelangeli et al. 2015). Environment‐dependent mate choice preferences can also drive fitness variation between populations or migrants, resulting in the evolution of reproductive isolation and, ultimately, speciation (Boughman 2002; Ritchie 2007).

Mate choice based on multiple cues may provide females with a means of accurately assessing male quality in changeable environments (Candolin 2003; Rundus et al. 2011) with each cue potentially reflecting a different aspect of male quality, or making additional contributions to a more accurate overall assessment (Johnstone 1996). There is increasing evidence that females use multiple cues when selecting a mate (Iwasa and Pomiankowski 1994; Candolin and Reynolds 2001; Candolin 2003 and references therein, Lehtonen et al. 2007; Lehtonen and Wong 2009) and may rely on different traits at particular stages of the decision‐making process (Candolin and Reynolds 2001). But when environments are subject to change, which traits should females rely on? Several studies have looked at the relative importance of multiple sexual cues under different ecological conditions, either in terms of male trait expression (Candolin et al. 2007; Lehtonen et al. 2015, 2016; Michelangeli et al. 2015; Candolin et al. 2016) or female mate choice (Hale and St. Mary 2007; Jävenpää and Lindström 2004; Candolin et al. 2007; Heuschele et al. 2009; Lehtonen and Wong 2009; Rundus et al. 2011; Candolin et al. 2016). However, we are not aware of any studies that have examined how both male trait expression and female mate choice for multiple cues are affected by switches in environmental conditions. Here, we assess how environmental fluctuation impacts on different types of male sexual cues (male phenotype vs extended phenotype (Dawkins 1989)) and whether such impacts affect the relative importance of multiple cues in female mate choice decisions.

Three‐spine sticklebacks are an ideal species with which to experimentally test how variable environments affect sexual selection on multiple sexual traits and the preferences for these traits. The three‐spine stickleback (*Gasterosteus aculeatus*) is a small, nest‐building fish found in a wide range of marine and freshwater habitats throughout the northern hemisphere. Male sticklebacks build nests by gluing together sediment and collected vegetation to form a mat that then functions both as a focus for courtship and a receptacle for eggs (Wootton 1976). During courtship males lead receptive females to their nest which the female inspects by pushing her snout into the nest entrance, after this the female may leave or enter the nest to spawn. Once a male has eggs in his nest he enters a parental phase during which he defends the nest from predators and competitors, fans developing embryos with oxygenated water and removes unfertilized, dead, and diseased embryos (Wootton 1984). Through the incubation period the male also modifies the structure of the nest to allow greater water transfer as embryos develop and oxygen consumption increases (Wunder 1930). Fish nest structure is intimately linked to the survival of offspring through its impact on oxygen availability (Jones and Reynolds 1999a; Green and McCormick 2004) and protection from predators (Wong et al. 2008), and therefore nest behaviors are expected to be under strong natural selection (Barber 2013). Indeed the nests of three‐spine sticklebacks show marked variation in structure between populations (Rushbrook and Barber 2008), ecotypes (Ólafsdóttir et al. 2006; Raeymakers et al. 2009), and individuals (Kraak et al. 1999; Barber et al. 2001). Such variation is likely due to both genetic differences between males (Rushbrook et al. 2008) and environmental differences between nest locations (e.g., flow regime (Rushbrook et al. 2010), and substrate types (Ólafsdóttir et al. 2006)). Female sticklebacks have been shown to choose nests dependent on their location (Kraak et al. 1999; Blais et al. 2004; Bolnick et al. 2015) and amount of decoration (Östlund‐Nilsson and Holmlund 2003) and it is expected that by assessing nest structure and location females may obtain information not only on the suitability of the nest as a receptacle for eggs but also on male quality because nest building is energetically expensive (Stanley 1983; Wootton 1985, 1994) and condition‐dependent (Barber et al. 2001; Rushbrook et al. 2008), meaning that nest‐building is also under strong sexual selection.

To determine how environmental fluctuations influence both male sexual cues and female mate choice for these cues, we manipulated dissolved oxygen (DO) level at two stages in the males’ reproductive cycle. First, males were exposed to either high or low DO conditions during the nest building phase. Once a male's nest was complete, he was then exposed to a second DO treatment in which we conducted mating trials (i.e., during the courtship and spawning period). Fluctuations in DO levels in the breeding habitats of male sticklebacks occur in nature as a consequence of the decomposition of vegetation, nutrient input, and changes in flow rate and may be exacerbated in locations subject to anthropogenic nutrient inputs (Walton et al. 2007). DO levels also fluctuate on a daily and seasonal basis, as a result of their correlation with temperature. Given that oxygen availability is one of the most important factors influencing fish embryo development (Rombough 1988), responding to changes in DO levels is likely to have consequences for offspring survival and growth (Jones and Reynolds 1999a; Lissåker et al. 2003). The aims of the study were (1) to determine whether males adjust the structure of their nests in response to varying DO, (2) to determine whether females evaluate nest characteristics in their mate choice decisions and whether this choice is environmentally dependent and (3) to examine how mate choice for an extended nest phenotype compares with mate choice for a males own phenotype under varying environmental conditions. Since nest structure is linked to offspring performance we predicted that males would adjust their nest structure to maximize offspring fitness and that females would choose to mate with males that have built nests best‐suited to the environmental conditions under which they are being assessed. Previous observations of males actively reducing nest compactness during the egg incubation period (Wunder 1930) suggest that compactness does play an important role in determining oxygen transfer to embryos in the nest and, hence, that less tightly structured nests would be preferred by females under low DO conditions. In addition, we predicted that environmentally dependent preferences are likely to evolve for traits whose function is dependent on the surrounding habitat but not for traits which are likely to signal the same thing regardless of the environment they are found in.

## Material and Methods

### ANIMALS AND HUSBANDRY

Adult three‐spine sticklebacks were caught in minnow traps from Carsington Reservoir, U.K. (53°3′30″N 1°37′50″W), transferred to aquarium facilities at the University of Leicester and held in single sex groups until they attained breeding condition. We used standard in vitro fertilization techniques (Barber and Arnott 2000) to generate full sibling clutches of fertilized eggs, each of which were placed in a plastic tea strainer and aerated from below in a 1L hatching tank. Newly hatched fry were fed infusoria for several days and then switched to a diet of *Artemia* spp. nauplii. After 6 weeks these juvenile fish were transferred to mixed family group tanks within a filtered, aerated recirculating aquarium system. They were fed brine shrimp (*Artemia* spp.) nauplii and bloodworm (*Chironomus* spp.) larvae daily and were subjected to natural seasonal temperature and light regimes to bring fish into reproductive condition the following Spring (Baggerman 1985). Prior to use in experiments, these lab‐bred males and females (identified by the presence or absence of breeding coloration) were separated into single sex tanks.

### EXPERIMENTAL SETUP

To determine the effect of DO on reproductive behavior (nest construction and mate choice) we employed a two‐phase experimental design where males were first exposed to one DO treatment (low/high) during the nest building period and then–‐once the nest was completed–‐exposed to a second oxygen treatment (low/high) in which mating trials took place. The number of males exposed to each of the four possible treatment combinations was: high→high *N* = 18, high→low *N* = 15, low→low *N* = 14, low→high *N* = 14, with sample sizes constrained by the number of males and aquaria available. High and low DO treatments were created by bubbling compressed air or nitrogen gas (respectively) into the water through an air stone (following Lissåker et al. 2003). DO levels were monitored twice daily using an oxygen meter (YSI 550A, calibrated daily) and were maintained at 30–40% air saturation in the low DO treatment (equivalent to an O_2_ level of 2.9–3.9 mg/L at 16–18°C), and 90–100 % air saturation in the high DO treatment (equivalent to an O_2_ level of 8.7–9.7 mg/L at 16–18°C). These values are within the range that these fish would experience in the wild; sticklebacks are tolerant of polluted water with relatively low oxygen levels (Katsiadaki et al. 2002; Sneddon and Yerbury 2004), and often inhabit bodies of water that experience severe declines in oxygen levels (Walton et al. 2007). These values have also been used successfully in previous studies on fish parental care behavior (Jones and Reynolds 1999a, b, c; Lissåker et al. 2003).

Prior to being placed in treatment aquaria (17.5 × 32 × 17 cm), males that showed nuptial coloration were weighed and measured (wet mass recorded to 0.001 g and standard length recorded to 0.01 mm, respectively). Aquaria were set up with two types of substrate, sand and aquarium gravel, each covering one half of the base and were held under a 16 hours light: 8 hours dark photoperiod and temperature of 17 ± 1°C, with fish fed bloodworm daily. After introduction to the aquaria, males were given 6 hours to acclimate to their treatment conditions before 400, 5 cm‐long black polyester nesting threads were added. One day later, and on each day thereafter, a gravid female housed in a (glass jar with a mesh top) was placed in the center of each male's tank for 20 minutes to encourage nesting.

Males were checked daily for the presence of a nest. Once a nest was observed, males were allowed 4 days to complete its construction, during which time daily presentations of gravid females in a glass jar continued. At the end of day four, we removed all unused nesting material and photographed the nest in situ (using a tripod‐mounted Fuji Finepix s9600 digital camera) for subsequent analysis of nest structure (see below). After photographing the nest, the second DO treatment level was applied to individual aquaria by bubbling either air or N_2_ through an air stone, and males were given 2–6 hours to acclimate to the new conditions before a free‐swimming gravid female was introduced. Courtship and mating behavior were then recorded under this second DO treatment level (see below).

### QUANTIFYING NEST STRUCTURE

Male nest structure was quantified at two time‐points in the experiment. Quantification was via a measure of nest compactness, determined from in situ photographs taken before spawning (i.e., still under initial DO treatment conditions) and after spawning (i.e., following the introduction of a female, mating, and spawning under the second DO treatment level). Only males that spawned were used in this analysis, with males that did not spawn with the first female introduced being presented with additional females until spawning did occur. Four males did not spawn at all, meaning that final sample sizes for this analysis were: high→high *N* = 16; high→low *N* = 15, low→low *N* = 14; low→high *N* = 12. Nest compactness was calculated as the bulk area of the nest divided by the total area of the nest (i.e., the proportion of nest area through which the basal substratum could not be seen) and provides a measure of nest density (for a detailed explanation of measurement, see Barber et al. 2001). The change in nest compactness recorded between these two time points (corresponding to before spawning and one day after spawning, as well as to a change in DO level for half of the males) provided information on how males adjust nest structure in response to both spawning and changes in oxygen levels.

### MATING TRIALS

Male courtship and mating success were assessed using “no choice” mating trials, which are routinely used in studies looking at mating preferences in sticklebacks (Head et al. 2009; see Nagel and Schluter 1998 for justification of this method). Gravid females were identified by gently squeezing their abdomen to confirm the presence of ripe eggs in the oviduct. They were then transferred to holding aquaria with equivalent experimental DO levels and allowed to acclimate for a period of 2–6 hours. Individual females were then transferred to a nesting male's aquarium and behavioral observations made for the following 10 minutes, or until the female had spawned in the nest. For males that underwent multiple mating trials to enable comparisons of nest structure before and after spawning, (see above), data from only the first mating trial were used in mate choice analyses. Five trials were excluded from the analysis of courtship because females were overly responsive (i.e., spawned without being led to the nest or followed the male prior to courtship), resulting in final sample sizes of: high→high *N* = 17; high→low *N* = 12; low→low *N* = 14; low→high *N* = 12. Behavior was recorded from approximately 2 m in front of the aquarium using a notebook PC with event recording software (Observer, Noldus Information Technology, Lessburg, VA, USA). Male courtship behaviors recorded included the frequency of zig‐zagging, biting, and nest gluing, as well as the frequency and duration of both nest tending and nest fanning (described in Rowland 1989). We also recorded whether or not the trial ended in a successful spawning, which we defined as the male swimming through the nest after the female deposited her eggs. For all male courtship traits we calculated the rate of each of the courtship behaviors over the duration of the trial, to account for trials that were terminated early due to the female entering the nest before 10 minutes was over.

### STATISTICAL ANALYSIS

The influence of DO on nest structure was investigated using a general linear model (GLM) with a Gaussian error structure. In the first instance, we tested how DO level during nest building affected nest structure by including nest compactness before spawning as our response variable and a male's first DO treatment as a fixed factor. We then tested how males changed their nest structure in response to receiving eggs in their nest and changing DO conditions by including the difference in nest compactness before and after spawning as the response variable and both the first and second DO treatments a male experienced as fixed factors. We analyzed our data using this two‐step approach because as nests measured before spawning had not yet been exposed to a second DO treatment, it was not possible to consider the data from the two time points in a single repeated measures analysis. We included male standard length as a covariate in all of these analyses, since male size has the potential to influence nest structure (Rushbrook et al. 2008; Wong et al. 2012).

The rate at which males performed courtship behaviors were all highly intercorrelated, most likely linked to variation in circulating levels of 11‐ketotestosterone among males (see Macnab et al. 2011). We therefore combined these behaviors into a single measure (“courtship”) using principal components analysis (PCA). All measured components of male courtship loaded strongly and positively on PC1 (apart from biting, which loaded negatively), with PC1 explaining 60.85% of the variation in male courtship data (electronic Table S1). To investigate the effects of DO level on male courtship behavior we then used GLM with a Gaussian error structure. Our response variable was the factor score of each male on PC1 derived from the PCA described above, and the first and second DO levels that a male experienced were fixed factors in the model, with male standard length included as a covariate.

To investigate how DO conditions affected female choice for males and their nests, we conducted a GLM with a binomial error structure, using the binary measure of whether or not a female spawned in a male's nest during the second DO treatment level as our response variable. Each male's first and second DO treatment levels were included as categorical factors, with nest compactness and “male phenotype” included as continuous factors. Because our previous analysis of the effects of DO level on male courtship behavior revealed male courtship rate and male length were highly correlated we combined these variables into a single measure of “male phenotype,” using PCA. In this analysis PC1 (male phenotype) accounted for 77.1% of the variance in the data, and both measures included in the PCA loaded strongly and positively on this vector (male courtship = 0.878, male length = 0.878).

In all analyses we included up to two‐way interactions and then dropped nonsignificant interaction terms to allow interpretation of main effects (Crawley 2007). All analyses were conducted in SPSS version 20.

## Results

### THE EFFECT OF DO ON NEST CONSTRUCTION

**Figure 1:**
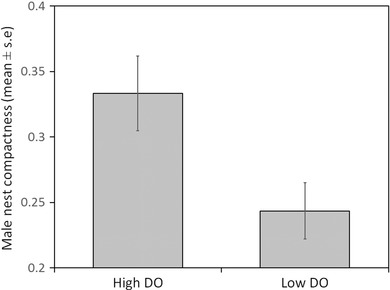
Effect of dissolved oxygen (DO) level on the structure of nests built by male three‐spine sticklebacks (*Gasterosteus aculeatus*). Nest structure is quantified in terms of the compactness of the nest material (bulk area of nest divided by total nest area) and relates to the structure observed at the end of the initial DO treatment exposure.

**Figure 2:**
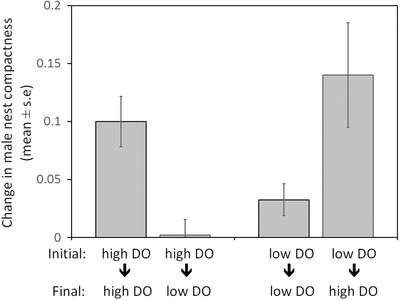
Effect of spawning and a change in dissolved oxygen (DO) level on the nest structure of male three‐spine sticklebacks (*Gasterosteus aculeatus*). Positive values indicate that nest compactness increased.

Males built more compact nests under high DO conditions than under low DO conditions (Fig. 1, F_(1,54)_ = 5.809, *P* = 0.019). These results were not dependent on male size (F_(1,54)_ = 0.031, *P* = 0.860) and the interaction term was not significant (F_(1,53)_ = 0.009, *P* = 0.924). In addition, males that experienced high DO after spawning increased the compactness of their nests, while those that experienced low DO after spawning did not (F_(1,46)_ = 17.842, *P* = 0.000) regardless of the DO conditions experienced during nest building (F_(1,46)_ = 2.555, *P* = 0.117) (Fig. 2). Again, these results were not dependent on male size (F_(1,46)_ = 0.297, *P* = 0.588) or any of the interaction terms (all *P* > 0.272).

### THE EFFECT OF DO ON MALE COURTSHIP

**Figure 3:**
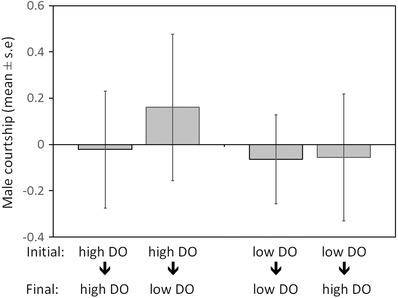
Effect of dissolved oxygen (DO) level on the courtship behavior of male three‐spine sticklebacks (*Gasterosteus aculeatus*). Male courtship is the first principal component of seven common stickleback courtship behaviors (see text). Loadings of these behaviors on PC1 are given in Table S1.

**Figure 4:**
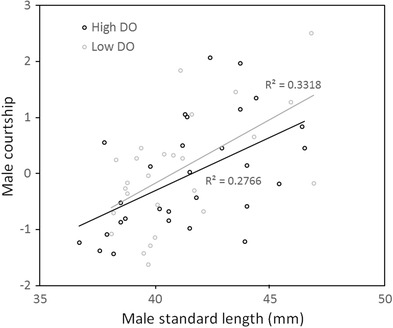
Relationship between male standard length and courtship exhibited by male three‐spine sticklebacks (*Gasterosteus aculeatus*) under high and low dissolved oxygen (DO) levels. Male courtship is the first principal component of seven common stickleback courtship behaviors (see text). Loadings of these behaviors on PC1 are given in Table S1.

Neither the first nor second DO treatment experienced by a male affected his courtship level (first DO level: F_(1,51)_ = 0.004, *P* = 0.948; second DO level: F_(1,51)_ = 0.534, *P* = 0.468) (Fig. 3). However, there was a strong effect of male size on courtship behavior (F_(1,51)_ = 21.658, *P* < 0.000), indicating that large males court more than small males (Fig. 4).

### THE EFFECT OF DO ON FEMALE CHOICE FOR MALES AND NESTS

**Figure 5:**
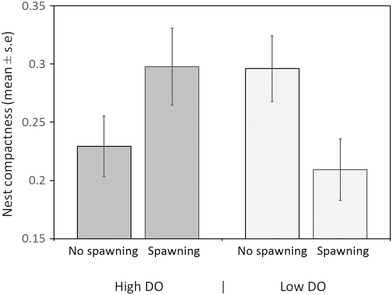
Effect of dissolved oxygen (DO) level on the degree of nest compactness eliciting a spawning response from female three‐spine sticklebacks (*Gasterosteus aculeatus*).

**Figure 6:**
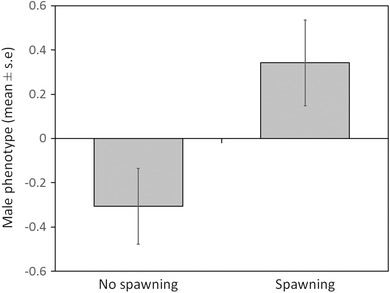
Effect of “male phenotype” on the decision by female three‐spine sticklebacks (*Gasterosteus aculeatus*) whether or not to spawn. Male phenotype represents the first principal component of male courtship and male standard length (see Methods).

Female mate choice based on nest compactness differed depending on the DO level under which the female was choosing (nest compactness × second DO interaction: χ^2^_(1)_ = 5.281, *P* = 0.022). Females spawning under high DO preferred nests that were more compact than females spawning under low DO (Fig. 5). Female mate choice based on male phenotype, on the other hand, was the same across treatments, with females always preferring large males with high levels of courtship (Fig. 6: χ^2^_(1)_ = 7.227, *P* = 0.007). The DO treatment under which a male built his nest did not affect female mate choice (χ^2^_(1)_ = 0.009, *P* = 0.923). Nor did any other interactions (all dropped from the model at *P* > 0.264).

## Discussion

How females assess multiple cues when choosing a mate in variable environments is key to understanding how these traits evolve and how animals cope with changing environments. Here, we show that male sticklebacks exhibited plasticity in their extended phenotype, building more compact nests in high DO than in low DO conditions, independent of male body size. Moreover, males exhibited within‐individual plasticity in their extended phenotype even over short timescales, modifying nest structure in response to rapid environmental changes. With regards to how environmental fluctuations influenced female mate choice, DO level affected female nest preferences, but not female preferences for male characteristics. Under high DO conditions females preferred more compact nests than they did under low DO conditions. However, under both conditions females preferred larger males with high courtship rates. We found no effect of DO conditions on the courtship behavior of males, but instead courtship was strongly related to male size, with large males courting more vigorously. Our results offer unique insight into the interactive effects of multiple sexual cues in variable environments in terms of their impact on both male behavior and female mate choice.

Male sticklebacks are known to adjust their nest structure in response to the increasing oxygen demands of developing embryos (Wunder 1930) and in response to changing water flow regimes, building more elongate nests under high flow conditions (Rushbrook et al. 2010). Here, we demonstrate that male sticklebacks not only build nests with different structures in response to DO, but also that they adjust nest structure over short timescales in response to changes in the DO content of the water. The previous observations that males modify nest structure in response to the increasing oxygen demands of developing embryos by loosening nest material and perforating the structure (Wunder 1930), combined with the fact that male courtship was not influenced by DO level, support the view that differences in nest compactness observed in this study between high and low DO conditions were a deliberate architectural choice by males, rather than being attributable to physiological constraints imposed by energetic demands of the low DO treatment. The assertion that results were not driven by any potential physiological constraints on the male's ability to build a nest under low DO is further supported by the fact that males in high DO conditions and whoe were therefore not under pyhsiological constraints at the building stage still opted to amend there nest structure when transferred to low DO conditions. Furthermore, the fact that females selected for different nest structures under the two DO treatments, actually preferring the “looser,” easier‐to‐build nest structures under low DO conditions, suggests that attempting to control for potential physiological constraints would not have had any impact on the study results.

Nest quality is typically tightly linked with offspring fitness in birds (Ricklefs 1969; Webb 1987) and in fishes (Barber 2013 and references therein), and therefore adjusting nest structure to suit environmental conditions is expected to increase a male's nesting success. Within‐individual variation in the expression of an extended phenotype over short timescales represents an important finding in terms of understanding the expression of sexually selected traits and also in terms of highlighting a neglected component of variation (within‐individual as opposed to between) (Griffith and Sheldon 2001). Accounting for within‐individual plasticity is critical to understanding the evolutionary potential of extended phenotypic traits under conditions of increased environmental heterogeneity that arise as a result of human‐induced, rapid environmental change (Price et al. 2003; Sih et al. 2011).

In the current study, body size did not mediate the response of individual males to environmental conditions. Differences in nest structure observed between initial high and low DO conditions and the alteration of nest‐structure in response to a change in DO levels were both independent of body size. This result contrasts with recent studies on sand gobies that show that male body size mediated the effects of turbidity (Lehtonen et al. 2015) and salinity (Lehtonen et al. 2016) on nest structure. The difference between our results and those of these previous studies may arise from differences in how the manipulated ecological parameter alters the costs and benefits of different nest structures and how these relate to body size. In the case of the sand gobies, differences in water turbidity influence perception of predation and large and small males have been shown to respond differently to the threat of predation in a reproductive context (Wong et al. 2009). Similarly, large and small males may respond differentially to increasing salinity levels, due to the varying levels of osmotic stress imposed on different body sizes and/or the size‐dependent abilities of male sand gobies to protect embryos from infection at lower salinities (Lehtonen et al. 2016). The lack of a size‐dependent effect on nest structure or the adjustment of nest structure, seen here, suggests that the costs and benefits incurred by small and large males in different DO conditions either do not differ (Lehtonen et al. 2015) or that the benefits associated with increased offspring survival far outweigh any potential size‐dependent costs of nest building.

But how does selection due to mate choice act on extended phenotypes in heterogeneous environments? Females choosing under low DO conditions preferred less compact nests, potentially because loosely constructed nests facilitate greater water transfer and thus oxygen exchange under low DO levels (Jones and Reynolds 1999a), whereas in high DO conditions they preferred more compact nests, potentially due to better protection against predators and intruders (Sargent 1982; Jones and Reynolds 1999c). Most previous research looking at mate choice for extended phenotypes has focused on extended phenotypes that act as signals (i.e., that have evolved to function in transmitting information about the builder (Schaedelin and Taborsky 2009)), for instance the bowers of bowerbirds (Borgia 1985; Endler et al. 2014) and cichlids (Mitchell et al. 2014; Jordan et al. 2016). These studies generally show directional female preferences for exaggerated condition‐dependent structures that reflect male quality (Borgia 1985; Jordan et al. 2016). Previous studies of nest building in sticklebacks have found that aspects of nest structure (including nest compactness) are condition‐dependent (Stanley 1983; Wootton 1985, 1994; Barber et al. 2001), suggesting that nests may be used by female sticklebacks as an honest signal of male quality (Barber et al. 2001). Here, we found that nest compactness was unrelated to male phenotype. However, nests could still signal male quality to females if high quality males are better at matching their nests to the environment and adjusting them quickly and effectively in response to environmental change. In which case, nest compactness would not only provide females with information on the suitability of the nest for rearing offspring, but also information about the male building the nest. This remains to be tested.

Unlike female mate choice for nest structure, female mate choice for male phenotype did not depend on the environmental context. This is likely because, in our study, male phenotype is a reliable proxy for overall male condition. As in many other taxa (Andersson 1994; Cothran 2008), large male sticklebacks, with high levels of courtship have been shown to have higher reproductive success (Rowland 1989; Jamieson and Colgan 1989; Kraak et al. 1999; Wong et al. 2012). Choosing large, vigorous males is therefore likely to benefit females in a broad range of environmental contexts. Differences between the way females assess nest structure and male phenotype suggest that females evaluate the reliability of both cues, taking into account the potential effects of environmental heterogeneity. Furthermore, our results suggest that the effect of temporal environmental fluctuations (as opposed to distinct alternatives in environmental conditions previously studied in this context for example turbid versus nonturbid habitat) may reduce directional sexual selection for traits that are environmentally‐dependent and strengthen directional selection for condition‐dependent traits. This has the potential to create a tug of war between natural and sexual selection in terms of creating greater mating opportunities for males with overall higher condition, but without necessarily greater levels of local adaptation, leading to a reduction in the rates of adaptation to prevailing local environmental conditions (Servedio and Bürger 2014). The overall effect of greater environmental variability could therefore be to slow rates of population differentiation, and hence rates of speciation. Alternatively, where synergies exist between the two types of traits, the overall effect may be to enhance mean fitness levels within the population and reduce variation in condition‐dependent traits, promoting the evolution of these traits and rates of speciation (Lorch et al. 2003). With the future likely to be characterized by increasing environmental variability as a consequence of anthropogenic impacts (Easterling et al. 2000; Field et al. 2012), clarifying the implications of these potential changes on population level genetic diversity, population viability, and rates of species’ evolution (Price et al. 2003; Higginson and Reader 2009; Ingleby et al. 2010; Candolin et al. 2015, 2016) will be an important next step.

Associate Editor: D. Shuker

Handling Editor: M. Servedio

## Supporting information


**Table S1**. Vector loadings on the first principle component of courtship behaviours performed by male three‐spined sticklebacks (*Gasterosteus aculeatus*) during experimental mate choice trials.
