# Supplementary material for: Environmental change mediates mate choice for an extended phenotype, but not for mate quality
Source: Evolution. 2016 Nov 2;71(1):135–44. doi: 10.1111/evo.13091 (PMC5298037; doi:10.1111/evo.13091)
Supplement: Supplementary file 1 — Table S1. Vector loadings on the first principle component of courtship behaviours performed by male three‐spined sticklebacks (Gasterosteus aculeatus) during experimental mate choice trials. [file EVO-71-135-s001.docx]

Supplementary material: M.L. Head, R.J. Fox, I. Barber. “Environmental heterogeneity mediates mate choice for an extended phenotype, but not for mate quality”

Table S1. Vector loadings on the first principle component of courtship behaviours performed by male three-spined sticklebacks (*Gasterosteus aculeatus*) during experimental mate choice trials. PC1 explained 60.85% of the variation in male courtship behavior.

| Rate of male courtship behaviours | Loading on PC1 |
| --- | --- |
| Zig-zagging | 0.762 |
| Biting | -0.585 |
| Tending frequency | 0.895 |
| Tending duration | 0.863 |
| Fanning frequency | 0.836 |
| Fanning duration | 0.767 |
| Gluing of nest structure | 0.710 |
